# Salutary effects of moderate but not high intensity aerobic exercise training on the frequency of peripheral T-cells associated with immunosenescence in older women at high risk of breast cancer: a randomized controlled trial

**DOI:** 10.1186/s12979-022-00266-z

**Published:** 2022-03-23

**Authors:** Grace M Niemiro, Adriana M Coletta, Nadia H. Agha, Preteesh Leo Mylabathula, Forrest L. Baker, Abenaa M Brewster, Therese B Bevers, Enrique Fuentes-Mattei, Karen Basen-Engquist, Emmanuel Katsanis, Susan C Gilchrist, Richard J. Simpson

**Affiliations:** 1 Department of Pediatrics, The University of Arizona, Tucson, Arizona USA; 2 The University of Arizona Cancer Center, Tucson, Arizona USA; 3 Department of Health and Kinesiology, The University of Utah, Salt Lake City, Utah USA; 4 Cancer Control and Population Sciences Program, Huntsman Cancer Institute, Salt Lake City, Utah USA; 5 Department of Health and Human Performance, University of Houston, Houston, Texas USA; 6 School of Nutritional Sciences and Wellness, The University of Arizona, Tucson, Arizona USA; 7 Department of Clinical Cancer Prevention, The University of Texas MD Anderson Cancer Center, Houston, Texas USA; 8 Department of Radiation Oncology Clinical Research, The University of Texas MD Anderson Cancer Center, Houston, Texas USA; 9 Department of Behavioral Science, The University of Texas MD Anderson Cancer Center, Houston, Texas USA; 10 Department of Immunobiology, The University of Arizona, Tucson, Arizona USA; 11 Department of Cardiology, The University of Texas MD Anderson Cancer Center, Houston, Texas USA

**Keywords:** Physical activity, Myokines, Exercise immunology, β2 adrenergic receptor, Aging, Maximal oxygen uptake, Recent thymic emigrants

## Abstract

**Background:**

Immunosenescence is described as age-associated changes within the immune system that are responsible for decreased immunity and increased cancer risk. Physically active individuals have fewer ‘senescent’ and more naïve T-cells compared to their sedentary counterparts, but it is not known if exercise training can rejuvenate ‘older looking’ T-cell profiles. We determined the effects of 12-weeks supervised exercise training on the frequency of T-cell subtypes in peripheral blood and their relationships with circulating levels of the muscle-derived cytokines (i.e. ‘myokines’) IL-6, IL-7, IL-15 and osteonectin in older women at high risk of breast cancer. The intervention involved 3 sessions/week of either high intensity interval exercise (HIIT) or moderate intensity continuous exercise (MICT) and were compared to an untrained control (UC) group.

**Results:**

HIIT decreased total granulocytes, CD4+ T-cells, CD4+ naïve T-cells, CD4+ recent thymic emigrants (RTE) and the CD4:CD8 ratio after training, whereas MICT increased total lymphocytes and CD8 effector memory (EM) T-cells. The change in total T-cells, CD4+ naïve T-cells, CD4+ central memory (CM) T-cells and CD4+ RTE was elevated after MICT compared to HIIT. Changes in $\dot{\mathrm{V}}{\mathrm{O}}_{2\max }$ after training, regardless of exercise prescription, was inversely related to the change in highly differentiated CD8+ EMRA T-cells and positively related to changes in β2-adrenergic receptor (β2-AR) expression on CM CD4+ and CM CD8+ T-cells. Plasma myokine levels did not change significantly among the groups after training, but individual changes in IL-7 were positively related to changes in the number of β2-AR expressing CD4 naïve T cells in both exercise groups but not controls. Further, CD4 T-cells and CD4 naive T-cells were negatively related to changes in IL-6 and osteonectin after HIIT but not MICT, whereas CD8 EMRA T-cells were inversely related to changes in IL-15 after MICT but not HIIT.

**Conclusions:**

Aerobic exercise training alters the frequency of peripheral T-cells associated with immunosenescence in middle aged/older women at high risk of breast cancer, with HIIT (pro-senescent) and MICT (anti-senescent) evoking divergent effects. Identifying the underlying mechanisms and establishing whether exercise-induced changes in peripheral T-cell numbers can alter the risk of developing breast cancer warrants investigation.

**Supplementary Information:**

The online version contains supplementary material available at 10.1186/s12979-022-00266-z.

## Introduction

Immunosenescence is the term used to describe age-associated declines in the normal functioning of the immune system, which has been attributed to poor vaccination responses, low-grade inflammation and increased rates of infection and cancer in older adults [1]. The peripheral T-cell compartment is particularly susceptible to age-related changes, evidenced by a lower CD4:CD8 T-cell ratio, an expansion of highly differentiated/exhausted T-cells, fewer naïve T-cells due to thymic atrophy, and the reemergence of latent herpesvirus infections (e.g. cytomegalovirus) that can drive T-cell exhaustion [2–9]. These features of immunosenescence are also correlative of future cancer occurrence and are prominent in women newly diagnosed with breast cancer [10, 11].

Lifestyle factors have been revealed as a potential mitigator of immunosensence and reduced cancer risk [1]. Physical activity can lower breast cancer risk by 10-20% and is widely promoted for its positive effects on the immune system [12]. Randomized control trials in older adults have demonstrated that exercise training can improve immune responses to vaccination and lower chronic low grade inflammation [13–18]. Cross sectional studies have consistently reported ‘younger looking’ T-cell profiles in physically active compared to inactive individuals, even in middle age (e.g. 50-65yrs) [19]. This includes an increased CD4:CD8 T-cell ratio, fewer CD4+ and CD8+ T-cells exhibiting phenotypes associated with differentiation and exhaustion, and greater frequencies of naïve T-cells and recent thymic emigrants (RTEs) that are capable of responding to novel antigens [19]. Physically active older adults also have better control of latent herpesvirus infections and display higher serum levels of muscle-derived cytokines, particularly IL-7 and IL-15, which are known to promote thymic output and maintain peripheral T-cell numbers and function [19, 20]. However, the effects of exercise training on peripheral markers of T-cell senescence have so far yielded inconsistent results, possible because the majority of these studies involve healthy adults with no identified risk of disease [1].

Given the emerging evidence linking immunosenescence with breast cancer, it is possible that exercise training can lower risk by promoting favorable changes to the peripheral T-cell compartment. Regular exercise is hypothesized to purge exhausted/senescent cells from the T-cell repertoire through apoptosis, leaving ‘space’ for newly generated naïve T-cells to create a ’younger looking’ and more efficient immune profile in a process that is facilitated by the release IL-7 and IL-15 from active skeletal muscle [21–23]. However, very few randomized control trials have determined the effects of exercise training on peripheral T-cell profiles of middle-aged/older adults, and no study, to our knowledge, has focused these efforts towards individuals who are at high risk for developing cancer. It has also been purported that exercise improves immune surveillance and lowers tumor burden due to the frequent mobilization and redistribution of effector lymphocytes via a mechanism that is dependent on catecholamine activation of lymphocyte β2 adrenergic receptors(AR) [1]. As lymphocyte β2-AR sensitivity decreases with age [24, 25], there is a need to determine if exercise training will affect β2-AR expression on blood lymphocytes in individuals at high risk of cancer.

The aim of this randomized control trial was to determine the effects of 12-weeks of structured exercise training on the frequency of blood T-cell subsets associated with immunosenescence in a population of middle-aged/older women identified as being at high risk of developing breast cancer, and to correlate these with changes in serum levels of the muscle-derived cytokines (i.e. myokines) IL-6, IL-7, IL-15 and osteonectin. We also compared moderate intensity continuous exercise training (MICT) to high intensity interval training (HIIT) due to the surging popularity, reported health benefits and comparatively low time commitment of HIIT [26]. While there are reported health benefits of HIIT over MICT for several endpoints associated with cardiometabolic disease, few studies have determined the effects of HIIT on markers of immunity [38]. We report here that improvements in cardiorespiratory fitness after training are inversely associated with a change in the frequency of highly differentiated CD8+ T-cells and positively associated with changes in β2-AR expression on central memory subsets of CD4+ and CD8+ T-cells. The numbers of naïve and memory subsets of CD4+ T-cells, RTE’s and the CD4:CD8 T-cell ratio were increased or maintained with MICT but reduced with HIIT. These findings indicate that aerobic exercise training is capable of altering the frequency of the peripheral T-cell pool and that it might be better to advocate for MICT over HIIT when prescribing exercise to improve immunity in middle aged/older women at high risk of breast cancer.

## Results

### Participant demographics

Participant characteristics are listed in Table 1. Of note, no differences between participants were seen in resting heart rate, fitness levels, or resting blood pressure before the intervention.

**Table 1:** Participant Demographic data before and after the 12-week intervention: Demographic data for all participants who completed exercise and blood sample testing before (Pre) and after (Post). Data are mean (SD) with bolded p-values indicating *p*<0.05

		Pre	Post	Within Subjects p-value	|Hedge’s *g|*
**Weight (kg)**	HIIT	84.46 (24.80)	83.69 (24.84)	0.12	0.03
	MICT	81.84 (13.25)	81.2 (13.12)	0.16	0.05
	UC	77.29 (10.14)	77.11 (9.52)	0.84	0.02
**BMI (kg/m**^**2**^**)**	HIIT	32.08 (9.04)	31.80 (9.12)	0.15	0.03
	MICT	31.03 (8.66)	31.97 (5.28)	0.16	0.05
	UC	29.83 (2.56)	29.76 (2.19)	0.84	0.03
**Resting HR (BPM)**	HIIT	71.00 (9.04)	67.2 (8.85)	0.86	0.41
	MICT	64.00 (20.93)	69.45 (9.16)	>0.99	0.19
	UC	72.64 (10.28)	69.64 (13.53)	>0.99	0.24
$\dot{\mathbf{V}}{\mathbf{O}}_{\mathbf{2}\mathbf{\max }}$ **(ml/kg/min)**	HIIT*	**19.00 (3.10)**	**21.9 (3.73)**	**0.0016**	**0.81**
	MICT	18.16 (5.99)	21.4 (4.09)	0.14	0.33
	UC	19.30 (3.92)	19.7 (4.40)	>0.99	0.09
$\dot{\mathbf{V}}{\mathbf{O}}_{\mathbf{2}\mathbf{\max }}$ **(L/min)**	HIIT*	**1.56 (0.33)**	**1.73 (0.40)**	**0.003**	**0.44**
	MICT	1.46 (0.41)	1.63 (0.23)	0.14	0.41
	UC	1.48 (0.26)	1.49 (0.28)	0.83	0.04
**Systolic Blood Pressure (mmHg)**	HIIT	123.00 (11.77)	116.2 (7.86)	0.29	0.65
	MICT	118.19 (38.18)	122.45 (8.12)	0.34	0.63
	UC	120.82 (7.78)	118.91 (10.48)	>0.99	0.2
**Diastolic Blood Pressure (mmHg)**	HIIT	77.36 (8.90)	77.2 (4.02)	>0.99	0.02
	MICT	73.75 (24.36)	79.09 (2.88)	>0.99	0.25
	UC	77.64 (3.98)	75.27 (3.82)	0.87	0.58

### CD4 T cells are decreased with HIIT training, but not MICT

**Fig. 1:**
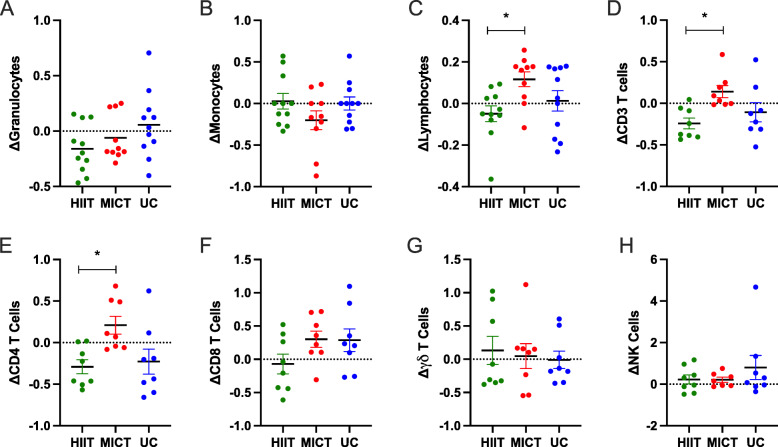
Lymphocytes, CD3 T cells, and CD 4 T cells are increased with MICT compared to HIIT. Between group comparisons of fold changes for leukocyte subsets before and after exercise training in older women. **A** Granulocytes, (**B**) Monocytes, and (**C**) Lymphocytes are depicted. **D** Total CD3 T cells are depicted. **E** γƍT cells, (**F**) Total CD4 T cells, (**G**) Total CD8 T cells, and (**H**) Total NK cells are depicted. Data are presented mean with *p<0.05

The HIIT group saw a decrease in overall granulocytes (*p*=0.05), CD4 T cells (*p*=0.01), and CD4:CD8 ratio (*p*=0.01; Table 2). The MICT group saw an increase in lymphocytes (*p*=0.01; Table 2). MICT had an increase in lymphocytes and CD3^+^ T cells after the intervention compared to HIIT (Fig. 1 C, 1D; *p*<0.05). CD4 T cells were decreased in the HIIT group compared to the MICT group (Fig. 1E; *p*<0.05). No other lymphocyte subsets were different between groups after the intervention. Changes in the proportions of these lymphocyte subsets were not different between groups (data not shown).

**Table 2:** The numbers of leukocyte subsets in peripheral blood before and after the 12-week intervention. Data are mean (SD) with bolded p-values indicating *p*<0.05

Leukocyte Subset		Pre (cells/µl WB)	Post (cells/µl WB)	Within Subjects p-value	|Hedge’s *g|*
**Granulocytes**	HIIT*	**4536.36 (1417.76)**	**3759.09 (1365.80)**	**0.05**	**0.54**
	MICT	4390 (800.28)	4105 (1154.81)	0.38	0.27
	UC	3640.91 (1457.19)	3868.18 (1996.28)	0.52	0.13
**Monocytes**	HIIT	431.82 (176.45)	413.64 (134.33)	0.62	0.11
	MICT	770 (715.77)	430 (153.12)	0.19	0.63
	UC	450 (159.69)	427.27 (123.21)	0.51	0.15
**Lymphocytes**	HIIT	1913.64 (320.23)	1822.73 (395.83)	0.21	0.24
	MICT*	**1675 (409.1)**	**1865 (484.22)**	**0.02**	**0.41**
	UC	1913.64 (462.11)	1886.36 (281.15)	0.8	0.07
**NK Cells**	HIIT	214.81 (86.69)	241.34 (101.59)	0.53	0.27
	MICT	241.07 (63.83)	298.37 (76.89)	0.14	0.6
	UC	138.05 (73.82)	180.03 (69.28)	0.12	0.55
**CD4 T cells**	HIIT*	**716.33 (252.13)**	**482.27 (191.75)**	**0.01**	**0.99**
	MICT	566.82 (265.99)	659.10 (325.2)	0.12	0.29
	UC	694.76 (349.47)	608.07 (344.74)	0.42	0.23
**CD8 T cells**	HIIT	273.99 (158.2)	256.86 (104.44)	0.64	0.12
	MICT	193.26 (134.67)	225.14 (112.57)	0.27	0.24
	UC	296.01 (231.72)	294.86 (222.39)	0.98	0.005
**γδ T cells (CD4**^**−**^**8**^**−**^**)**	HIIT	85.99 (101.84)	69.09 (62.24)	0.3	0.19
	MICT	33.54 (23.49)	29.71 (18.25)	0.67	0.17
	UC	56.26 (22.82)	59.70 (43.38)	0.73	0.09
**CD4:CD8**	HIIT*	**3.1 (1.25)**	**2.02 (0.68)**	**0.01**	**1.01**
	MICT	3.83 (2.03)	3.22 (1.45)	0.32	0.33
	UC	3.79 (3.34)	2.74 (1.81)	0.38	0.37

### CD4 naïve T cells and CD4 recent thymic emigrants are decreased with HIIT training

**Fig. 2:**
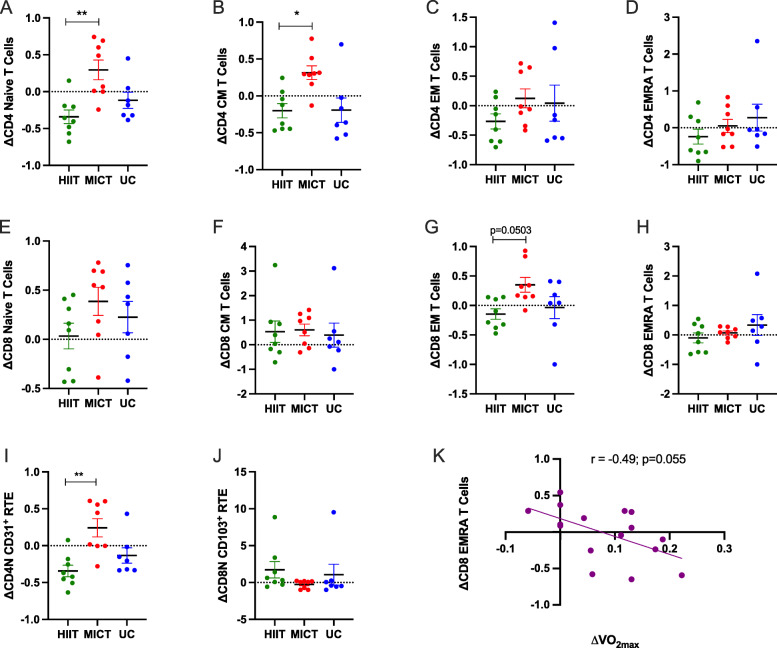
CD4 Naïve and Central Memory T cells are decreased with HIIT compared to MICT, and changes in fitness negatively correlate to changes in CD8 Effector Memory CD45RA+ T cells. Between group comparisons for differentiated T cell subsets before and after exercise training in older women. **A-D** CD4 Subsets [N=naïve, CM=central memory, EM=effector memory, EMRA=Effector Memory CD45RA^+^], (**E-H**) CD8 Subsets [N=naïve, CM=central memory, EM=effector memory, EMRA=Effector Memory CD45RA^+^], (**I**) CD4 Recent thymic emigrants (CD4N+CD31+), (**J**) CD8 Recent thymic emigrants (CD8N+CD103+). **K** Changes in fitness are negatively correlated to changes in CD8 EMRA T cells. Data are presented mean with *p<0.05

The HIIT group displayed a decrease in CD4 Naïve T cells (time effect *p*=0.02; Table 3) and CD4 recent thymic emigrants (RTE; time effect *p*=0.01; Table 3) after the intervention. The MICT group displayed a significant increase in CD8^+^ effector memory T cells (time effect *p*=0.03; Table 3) after training. All other differentiated subsets of CD4 and CD8 T cells were unaffected by training. CD4 Naïve T cells and CD4 central memory (CM) T cells were significantly decreased in the HIIT group compared to the MICT group (Fig. 2 A, 2B). There was a trend for an increase in CD8 effector memory (EM) T cells in the MICT group compared to HIIT (Fig. 2G). There was a significant decrease in CD4 RTE in the HIIT group compared to the MICT group (Fig. 2I). Of interest, in the exercise groups only (HIIT and MICT combined), there was a trend for a negative relationship between changes in $\dot{\mathrm{V}}{\mathrm{O}}_{2\max }$ and changes in (EMRA) CD8 T-cells (r=-0.49, *p*=0.055; Fig. 2 K). No changes in the proportions of these cell populations were found (data not shown). The levels of β2-AR expression (median fluorescence intensity; MFI) on lymphocyte subsets did not change after exercise training (Table 4).

**Table 3:** The numbers of CD4+ and CD8+ T-cell subsets in peripheral blood before and after the 12-week intervention. N=naïve, CM=central memory, EM=effector memory, EMRA= CD45RA^+^ Effector Memory, RTE=recent thymic emigrants. Data are mean (SD) with bolded p-values indicating *p*<0.05

T-cell Subset			Pre (cells/µl WB)	Post (cells/µl WB)	*p*	|Hedge’s *g|*
**CD4+**	**Naïve**	HIIT*	**264.03 (133.13)**	**163.46 (79.56)**	**0.02**	**0.87**
		MICT	148.70 (94.68)	178.94 (109.27)	0.17	0.28
		UC	190.23 (97.01)	177.37 (131.75)	0.6	0.1
	**CD4 Naïve CD31+ (RTE)**	HIIT*	**227.36 (105.46)**	**142.48 (62.28)**	**0.01**	**0.93**
		MICT	134.54 (86.43)	154.74 (95.08)	0.3	0.21
		UC	172.14 (90.48)	158.42 (123.98)	0.55	0.12
	**CM**	HIIT	225.62 (76.13)	173.93 (59.4)	0.07	0.72
		MICT	223.88 (107.66)	287.67 (134.81)	0.01	0.49
		UC	270.94 (176.45)	224.46 (178.24)	0.3	0.25
	**EM**	HIIT	191.09 (68.62)	132.26 (77.17)	0.05	0.76
		MICT	189.59 (90.57)	194.86 (90.08)	0.82	0.06
		UC	240.05 (161.12)	171.61 (119.61)	0.22	0.45
	**EMRA**	HIIT	39.71 (39.04)	22.26 (16.32)	0.28	0.55
		MICT	20.65 (12.36)	19.35 (10.55)	0.73	0.11
		UC	27.82 (20.55)	26.56 (21.02)	0.66	0.06
**CD8+**	**Naïve**	HIIT	98.83 (68.05)	95.93 (51.65)	0.87	0.05
		MICT	70.52 (68.04)	77.63 (49.88)	0.63	0.11
		UC	109.05 (112.75)	114.4 (98.08)	0.84	0.05
	**CD8 Naïve CD103+ (RTE)**	HIIT	1.87 (1.34)	6.46 (10.26)	0.22	0.59
		MICT	1.04 (0.43)	0.95 (0.49)	0.49	0.2
		UC	4.55 (4.96)	2.78 (3.38)	0.41	0.39
	**CM**	HIIT	37.31 (20.55)	44.46 (24.35)	0.6	0.3
		MICT	32.69 (20.5)	41.93 (16.85)	0.19	0.47
		UC	38.94 (21.15)	37.56 (30.14)	0.91	0.04
	**EM**	HIIT	93.01 (49.08)	74.95 (31.71)	0.1	0.26
		MICT*	**65.78 (40.84)**	**82.29 (45.1)**	**0.03**	**0.38**
		UC	98.29 (92.73)	87.16 (76.22)	0.57	0.31
	**EMRA**	HIIT	44.84 (40.85)	41.53 (45.96)	0.63	0.07
		MICT	24.28 (18.12)	23.29 (12.44)	0.67	0.06
		UC	49.74 (42.38)	55.74 (71.31)	0.82	0.1

**Table 4:** Relative β_2_-AR expression (MFI: median fluorescent intensity) in peripheral blood CD4+ and CD8+ T-cell subsets before and after the 12-week intervention. MICT and HIIT did not cause any changes in the expression (MFI; median fluorescence intensity) on lymphocyte subsets and differentiated T cell subsets. [CM=central memory, EM=effector memory, EMRA=Effector Memory CD45RA^+^] Data are mean (SD). No significant changes were found in response to the intervention (*p*>0.05)

Lymphocyte Subset			Pre (β2-AR MFI)	Post (β2-AR MFI)	*p*	|Hedge’s *g|*
**CD4 β2-AR+ T cells**		HIIT	20.77 (5.22)	23.46 (4.87)	0.23	0.5
		MICT	22.31 (7.44)	24.05 (4.98)	0.51	0.26
		UC	18.5 (6.92)	20.27 (6.07)	0.5	0.25
**β2-AR+ CD4**	**β2-AR+ Naïve**	HIIT	30.11 (9.90)	38.38 (15.33)	0.25	0.61
		MICT	33.54 (12.99)	33.36 (6.73)	0.97	0.02
		UC	29.19 (18.33)	28.99 (11.41)	0.97	0.01
	**β2-AR+ CM**	HIIT	22.09 (5.24)	25.49 (6.56)	0.12	0.54
		MICT	25.44 (7.95)	26.47 (4.95)	0.76	0.15
		UC	19.31 (6.33)	21.53 (5.27)	0.42	0.36
	**β2-AR+ EM**	HIIT	13.05 (2.99)	13.79 (2.29)	0.41	0.26
		MICT	13.07 (3.25)	15.09 (3.73)	0.14	0.54
		UC	12.92 (3.67)	13.09 (3.22)	0.9	0.04
	**β2-AR+ EMRA**	HIIT	17.78 (8.96)	26.48 (14.83)	0.24	0.67
		MICT	15.03 (5.12)	25.74 (10.15)	0.07	1.26
		UC	17.02 (9.21)	19.54 (14.54)	0.65	0.19
**CD8 β2-AR+ T cells**		HIIT	28.73 (11.19)	38.58 (21.61)	0.19	0.54
		MICT	36.15 (14.17)	39.18 (18.32)	0.66	0.17
		UC	23.87 (13.47)	128.81 (10.94)	0.38	0.38
**β2-AR+ CD8 T cells**	**β2-AR+ Naïve**	HIIT	28.98 (11.94)	41.57 (27.75)	0.23	0.56
		MICT	34.91 (18.50)	35.28 (22.56)	0.96	0.02
		UC	27.4 (20.74)	30.11 (16.36)	0.69	0.14
	**β2-AR+ CM**	HIIT	52.62 (18.98)	67.57 (27.14)	0.13	0.6
		MICT	74.54 (33.51)	86.42 (45.42)	0.57	0.28
		UC	43.86 (21.31)	49.47 (22.42)	0.5	0.5
	**β2-AR+ EM**	HIIT	13.96 (4.2)	17.80 (6.54)	0.2	0.66
		MICT	18.11 (7.14)	16.92 (4.78)	0.74	0.19
		UC	14.07 (2.65)	15.76 (4.75)	0.42	0.41
	**β2-AR+ EMRA**	HIIT	22.71 (13.02)	15.88 (6.1)	0.21	0.63
		MICT	18.20 (11.77)	21.75 (12.91)	0.07	0.27
		UC	16.72 (7.13)	12.85 (5.59)	0.27	0.56
**γδ T cells (Vδ2+) β2-AR+**		HIIT	13.63 (12.05)	17.58 (11.38)	0.42	0.32
		MICT	4.67 (8.83)	11.73 (6.4)	0.11	0.87
		UC	8.95 (8.51)	10.45 (6.71)	0.7	0.7
**NK Cells β2-AR+**		HIIT	36.29 (11.54)	34.89 (7.69)	0.78	0.13
		MICT	33.38 (9.22)	21.51 (6.37)	0.51	0.22
		UC	30.30 (9.23)	30.08 (7.61)	0.91	0.02

### Plasma myokines are related to changes in lymphocyte subsets and β2-AR expression

**Fig. 3:**
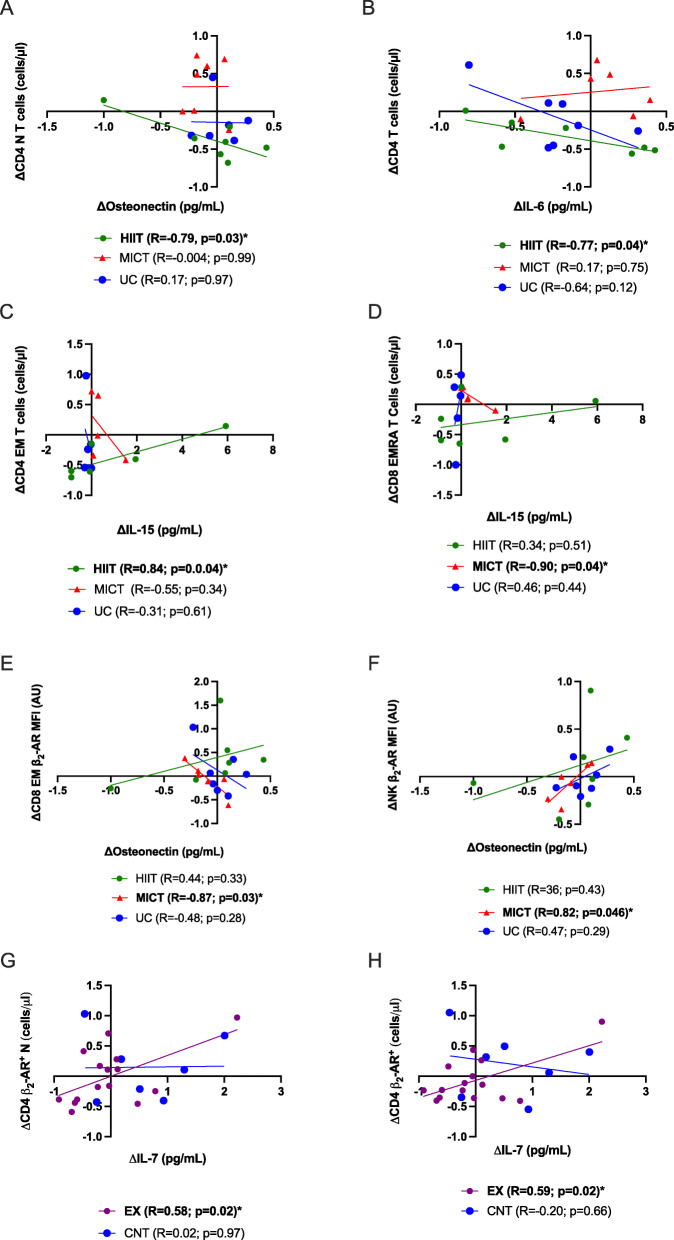
Changes in osteonectin, IL-6, IL-15 and IL-7, prominent myokines, are related to changes in immune cell subsets with exercise. **A, B** Changes in osteonectin and IL-6 were negatively related to changes in CD4 N T cells and CD4 T cells in the HIIT group only, respectively. **C,D** Changes in IL-15 were negatively related to changes in CD4 EM T cells in the HIIT group only; and negatively related to changes in CD8 EMRA T cells in the MICT group only. **E, F** Changes in osteonectin were negatively related to changes in β2-AR expression CD8 EM T cells and positively related to changes in the β2-AR expression on NK cells, respectively, in the MICT group only. **G, F** Changes in IL-7 were positively related to changes in β2-AR expression on CD4 N T cells and β2-AR expression on CD4 T cells in the exercise (HIIT+MICT; respectively) groups only. N=naïve, CM=central memory, EM=effector memory, EMRA=Effector Memory CD45RA^+^. Lines represent line of best fit and were analyzed by Pearson’s Correlation with *p*<0.05 considered statistically signification. *p<0.05

No changes were observed between groups for levels of circulating myokines IL-7, IL-1, IL-6, and osteonectin (supplementary Fig. 1) [27]. Changes in plasma osteonectin were negatively related to changes in CD4 naïve T cells in the HIIT group only (r=-0.79; Fig. 3 A); and changes in IL-6 were negatively related to changes in CD4 T-cells in the HIIT group only (r=-0.77, Fig. 3B). Changes in plasma IL-15 were postively related to changes in the number of circulating CD4 EM T cells in the HIIT group only (r=0.84; Fig. 3 C) and negatively related to changes in the number of circulating CD8 EMRA T cells in the MICT group only (r=-0.90; Fig. 3D). Changes in plasma osteonectin were negatively related to changes in β2-AR expression on CD8 EM T cells in the MICT group only (r=-0.87; Fig. 3E) and positively related to changes in β2-AR expression on NK cells in the MICT group only (r=0.82; Fig. 3 F). Changes in IL-7 were found to be positively related to changes in β2-AR expressing CD4 T cells (r=0.58; Fig. 3G) and β2-AR expressing CD4 naive T cells (r=0.59; Fig. 3 F) in the exercise groups (HIIT+MICT) only.

### Changes in $\dot{\mathrm{V}}{\mathrm{O}}_{2\max }$ are positively correlated with β2-AR expression levels on T-cell subsets

**Fig. 4:**
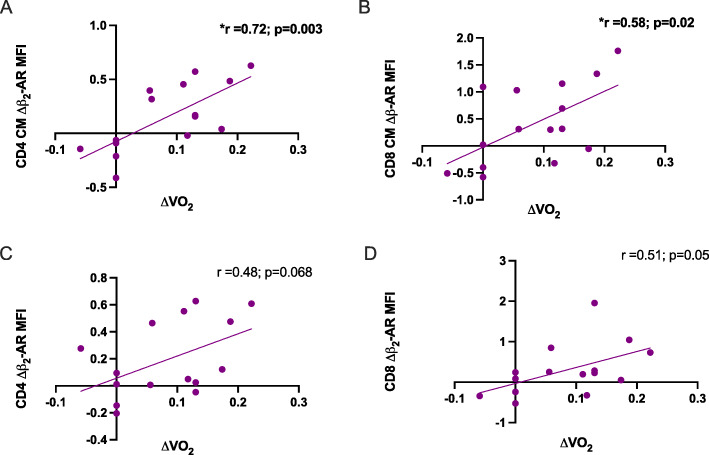
Changes in fitness are positively related to changes in β_2_-AR expression on CD4 CM and CD8 CM Differentiated T cells in HIIT and MICT groups combined. Changes in fitness in the exercise groups were positively related to changes in (**A**) CD4 CM β_2_-AR expression, (**B**) CD8 CM β_2_-AR expression, (**C**) CD4 β_2_-AR expression, and (**D**) CD8 β_2_-AR expression. [CM=central memory]. Lines represent line of best fit and were analyzed by Pearson’s Correlation with *p*<0.05 considered statistically signification. *p<0.05

When combining the HIIT and MICT groups together and comparing to changes in $\dot{\mathrm{V}}{\mathrm{O}}_{2\max }$, there were trends for a positive relationship between the Δβ2-AR MFI on CD4 CM T-cells (r=0.72, Fig. 4 A). Furthermore, this positive relationship was significant on CD8 CM T-cell Δβ2-AR MFI expression (r=0.58, Fig. 4B). Similar relationships were seen in the parent T-cell populations, with a positive trending relationship between the changes in $\dot{\mathrm{V}}{\mathrm{O}}_{2\max }$ and the Δβ2-AR MFI expression on CD4 T cells (r=0.48, *p*=0.05, Fig. 4 C) and Δβ2-AR MFI expression on CD8 T-cells (r=0.51, *p*=0.05, Fig. 4D). The untrained control (UC) group had negative relationships compared to the exercise groups (data not shown).

## Discussion

The aim of this study was to determine the effects of supervised aerobic exercise training and to compare two forms of exercise prescription (HIIT or MICT) on peripheral T-cell subsets indicative of immunosenescence in a population of older women at high risk of developing breast cancer. The main findings from this study were: (1) HIIT decreased total granulocytes, total CD4+ T-cells, CD4+ naïve T-cells, CD4+ RTE and the CD4:CD8 ratio after 12-weeks training, whereas MICT increased total lymphocytes and CD8 EM T-cells; (2) The change in number of total T-cells, CD4+ naïve T-cells, CD4+ CM T-cells and CD4+ RTE was elevated after MICT compared to HIIT; and (3) changes in $\dot{\mathrm{V}}{\mathrm{O}}_{2\max }$ after training, regardless of exercise prescription, was positively related to changes in β2-AR expression on CM subsets of both CD4+ and CD8+ T-cells, and tended to be negatively related to the change in CD8+ EMRA T-cells.

Immunosenescence increases risk of developing age-related diseases such as cancer [11]. Changes in the composition of peripheral T-cells are hallmark features of immunosenescence, with ‘older looking’ T-cell compartments (e.g. lower CD4:CD8 T-cell ratios, increased CD8 EMRA T-cells, and fewer naïve and RTE subsets of CD4+ and CD8+ T-cells) being predictive of poor immune responses to vaccination and all-cause mortality in older adults [28]. T-cell profiles associated with immunosenescence were identified in women recently diagnosed with breast cancer and there is emerging evidence that immunosenescence could precipitate cancer occurrence [29, 30]. Exercise is known to increase immune function over the lifespan and concomitantly reduce cancer risk, particularly breast, prostate, colorectal and lung cancer [22, 31]. While cross-sectional studies have provided evidence that exercise can mitigate age-related changes in the peripheral T-cell compartment [5, 19, 19, 21, 22], longitudinal studies are required for us to know if exercise can help rejuvenate an already acquired ‘older looking’ T-cell profile. For the first time, we show here that MICT, but not HIIT, positively alters the peripheral T-cell compartment toward a less senescent phenotype. After training, the change in numbers of total T-cells, CD4+ naïve T-cells, CD4+ CM T-cells and CD4+ RTE were elevated after MICT compared to HIIT. Remarkably, MICT and HIIT tended to evoke divergent effects on the peripheral T-cell pool, with those T-cell subtypes found to increase or be maintained after MICT had conversely decreased after HIIT. The finding that MICT but not HIIT increased the frequency of CD8+ EM cells in circulation could have important implications for anti-tumor immune surveillance as these are the CD8+ T-cell subsets that predominantly infiltrate human breast tumors [32].

Exercise interventions involving HIIT have been preferred over MICT due to the lower time commitment and comparable improvements in cardiorespiratory fitness and biomarkers of inflammation [33, 34]. However, the present findings indicate that HIIT may not be an effective form of exercise training to evoke positive changes in the frequency of the peripheral T-cell compartment as they relate to markers of immunosenescence and could actually promote a pro-senescent phenotype. For instance, HIIT reduced naïve CD4+ T-cell numbers by ~38% whereas MICT tended to increase naïve CD4+ T-cells by ~20%. Although IL-7 is known to promote thymic mass and correlates with the frequency of peripheral naïve T-cells and RTEs [35, 36], we surprisingly found no relationships between changes in serum IL-7 and RTEs despite observing a trend for serum IL-7 levels to decrease after HIIT but not MICT. We did find, however, that changes in IL-7 were positively related with changes in the number of circulating CD4 and CD4 naïve T cells expressing the β_2_-AR in both treatment groups (HIIT+MICT) but not the controls. It has been shown in mice that the β_2_-AR binds norepinephrine to generate Th1 cells that produce 2- to 4-fold more IFN-γ during an immune response [37]. It is possible, therefore, that IL-7 levels regulated by exercise can influence the generation of naïve CD4+ T-cells that are capable of differentiating into Th1 T-cells via norepinephrine but this requires further investigation. Additionally, HIIT reduced the total number of circulating granulocytes to near significant (*p*=0.05) levels whereas MICT did not, indicating a potentially greater anti-inflammatory response promoted by HIIT over MICT. Indeed, a recent pilot study reported improvements in neutrophil function after 10-weeks of HIIT in older adults identified as high risk of developing type 2 diabetes, although this study did not compare HIIT to MICT [38]. Collectively, these data underscore the importance of exercise, mode, intensity, duration and volume when it comes to prescribing exercise for immune and anti-inflammatory benefits in older adults, and in people at high risk of disease.

Although neither MICT or HIIT significantly altered the number of CD8 EMRA T-cells, we did find a near significant inverse relationship between the changes in absolute $\dot{\mathrm{V}}{\mathrm{O}}_{2\max }$ (ml/min) and number of CD8+ EMRA T-cells (*p*=0.055). We previously reported inverse relationships between $\dot{\mathrm{V}}{\mathrm{O}}_{2\max }$ and highly differentiated (e.g. KLRG1+/CD28-/CD57+) CD8+ T-cells associated with immunosenescence in healthy men [39]. These cross-sectional findings have been corroborated by other groups, in addition to the observation that $\dot{\mathrm{V}}{\mathrm{O}}_{2\max }$ is positively associated with the composition of naïve CD4+ and CD8+ T-cells and RTEs in peripheral blood [19, 35]. Collectively, these findings indicate that an exercise training program may have to evoke discernible changes in aerobic fitness to markedly lower the frequency of late-stage differentiated T-cells in the periphery. While previous longitudinal studies have failed to report discernible changes in the numbers of peripheral T-cells associated with immunosenescence [21, 22], we feel that the present study succeeded because of our rigorous experimental design (e.g. all exercise sessions were supervised and involved individualized training zone prescriptions). Moreover, our participants were at high risk of disease and mostly obese as well as being older. Many previous studies have focused on healthy individuals who might have less bandwidth for altering T-cell numbers with exercise training [21, 22].

A recent study reported a decrease in ‘senescent’ CD57+CD8+ T-cells and an increase in naïve CD8+ T-cells in peripheral blood following 6-weeks strength endurance training in older women seropositive to cytomegalovirus [40]. The positive shifts in T-cell frequency reported here and by Dinh et al., bolstered by previous randomized controlled trials showing increased immune responses to vaccination after a period of exercise training [41, 42], provide robust evidence that exercise training is capable of rejuvenating an already acquired senescent phenotype to evoke meaningful changes in overall immune function. Moreover, while evidence is beginning to show that patients newly diagnosed with cancer have immunosenescent profiles and that exercise can extend survival during treatment for breast cancer and other solid tumors [43, 44], whether these exercise-induced changes in the frequency of T-cell subsets indicative of immunosenescence can lower the risk of developing breast cancer remains to be determined. A recent long-term follow up study of >50,000 women found no relationship between circulating numbers of CD4+ or CD8+ T-cells and risk of developing breast cancer [45], although it is important to note that this study did not consider T-cell subset composition (e.g. naïve, CM, EM and EMRA) or functionality, which can be altered considerably without greatly affecting total CD4+ or CD8+ T-cell numbers [46–49]. It will also be important to consider how exercise-induced shifts in T-cell subsets can influence prognosis in breast cancer patients on active treatment and at different stages of disease. For instance, in patients with metastatic breast cancer, a higher frequency of circulating naïve CD4+ and CD8+ T-cells is associated with poorer prognosis in patients treated with high-dose paclitaxel but not cyclophosphamide containing regimens [50], while higher numbers of late-stage differentiated CD8+ T-cells have been associated with shorter progression-free survival and overall survival [30, 51].

The mechanisms by which exercise training can alter the composition of the peripheral T-cell compartment have not been fully determined. Exercise may limit the age-related expansion of late-stage differentiated T-cells by helping to exert better control over latent viral infections (e.g. CMV), or by progressively removing these cells by increasing their exposure to pro-apoptotic stimuli over time [39, 52]. Increased apoptosis of late-stage differentiated T-cells by exercise has been hypothesized to promote the mobilization of ‘new recruits’, facilitated by an increase in hematopoiesis and muscle-derived cytokines, such as IL-7, that can promote maintenance of thymic mass and increase production and development of naïve T-cells [21]. As previous studies found positive associations between plasma levels of muscle-derived cytokines such as IL-7 and IL-15 and numbers of naïve and RTE T-cells in the blood of older endurance trained athletes [1, 19, 53], we investigated here whether changes in T-cell frequency were associated with changes in the levels of circulating IL-7, IL-15, IL-6, and ostenectin after the 12-week training intervention. IL-15 is an important myokine for optimal memory T-cell responses [54], increases T-cell antitumor immunity [55], and general T-cell activation and function [56], and has been shown to be highly expressed in muscle [57]. IL-6, on the other hand, is thought to be an overall pro-inflammatory cytokine [58] responsible for increasing chronic morbidity and aging [59], but its release from skeletal muscle during exercise plays an anti-inflammatory role [60] and has also been shown to facilitate tumor infiltration of exercise-mobilized NK-cells [61–63]. Osteonectin is a myokine that has been shown to inhibit tumorigenesis in colon cancer and to also potentially play a role in repairing damaged skeletal muscle [64]. Here, we found that the change in CD4 T-cells and CD4 naive T-cells were negatively related to changes in IL-6 and osteonectin after HIIT but not MICT, indicating a potential role for osteonectin and IL-6 in the maintenance of peripheral naïve T-cells in response to exercise training. We also found that changes in CD8 EMRA T-cells were inversely correlated with serum IL-15 levels for the MICT but not the HIIT group. More research is needed to identify potential causative roles for these myokines in regulating the peripheral T-cell compartment after exercise training.

The β2-AR has been shown to play an important role in the activation, mobilization and redistribution of immune cells with an increased ability to infiltrate tumors in response to exercise. In murine models of cancer, exercise has been shown to promote CD8+ T-cell and NK-cell infiltration to tumors and suppression of tumor growth via a catecholamine and β2-AR dependent mechanism [63, 65, 66]. Potential increases in β2-AR expression with exercise training might help contribute to a more effective anti-tumor response. While neither MICT or HIIT affected β2-AR expression, we did find a positive association between the changes in absolute $\dot{\mathrm{V}}{\mathrm{O}}_{2\max }$ and β2-AR expression on the surface of CM subsets of both CD8+ and CD4+ T-cells. There was also a trend for a positive relationship between the changes in β2-AR expression on CD8+ CM T cells and plasma osteonectin levels. The β2-AR has also been shown to modulate memory CD8 T-cell function [67], making the β2-AR a potential target in mediating aging related immunosenescence via exercise training https://pubmed.ncbi.nlm.nih.gov/34302965/.

Despite this being the first randomized controlled trial to show that an aerobic exercise training intervention can positively alter the frequency of T-cell subsets indicative of immunosenescence in a population of older women at high risk of developing breast cancer, we do acknowledge several limitations. These include the small sample size, lack of an endpoint measure of global immune competency (e.g. systemic challenge with a vaccine or experimental antigen), and the correlative nature of several of our findings. We also did not control for dietary intake and focused solely on a population of women at high risk for breast cancer who were also overweight/obese. As adiposity is known to influence immune cell function and phenotype [68–71], our interpretations of these findings must be taken with caution when applied to the lean older adult population.

## Conclusions

Aerobic exercise training is capable of altering the frequency of peripheral T-cells associated with immunosenescence in older women at high risk of breast cancer with divergent effects seen for MICT versus HIIT. Increases in $\dot{\mathrm{V}}{\mathrm{O}}_{2\max }$ after training, regardless of exercise prescription, are associated with an increase in β2-AR expression on CM subsets of CD4+ and CD8+ T-cells and a reduced frequency of EMRA CD8+ T-cells. Further study is required to identify the mechanisms underlying the opposing effects of HIIT and MICT on the frequency of peripheral T-cells, their relationships with circulating myokines, and to determine if exercise-induced changes in immunity can alter the risk of developing breast cancer.

## Materials and methods

### Study design, participants, recruitment and intervention rocedures

This was a parallel group, randomized controlled trial that consisted of a 12-week supervised exercise training intervention. Study groups consisted of HIIT (*n*=8), MICT (*n*=8), and UC (*n*=8). This investigation was approved by the University of Texas MD Anderson Cancer Center Institutional Review Board. Details regarding eligibility criteria, recruitment and intervention procedures are reported elsewhere [27, 72].

Eligible participants were post-menopausal women who were overweight or obese, as defined by body mass index (≥ 25 kg/m^2^), who were considered at heightened risk of developing breast cancer due to an elevated Gail 5-year risk score (> 1.66%), lifetime risk score (> 20%), history of ductal or lobular atypia, or history of ductal or lobular carcinoma in situ (non-invasive breast cancer). Participants were recruited from the University of Texas MD Anderson Cancer Center, Clinical Cancer Prevention Center.

Participants assigned to HIIT and MICT presented at the Cancer Prevention Center three times per week and completed supervised treadmill exercise, as previously described [27]. Briefly, HIIT consisted of a 5-minute warm-up, four 4-minute high-intensity intervals, followed by a 3-minute active recovery interval for 33 min. The MICT workout was 41 min in length, ensuring HR stayed consistent during the whole session. Participants assigned to UC received educational material related to healthful diet and exercise habits at baseline, and monthly phone calls by study personnel pertaining to their healthy lifestyle goals. More thorough descriptions can be found in previous literature [27, 72].

### Assessment procedures

Assessment sessions, including fitness testing, were conducted at baseline, 6-weeks and upon completion of the 12-week intervention (end-of-study). Procedures are described in detail elsewhere [27, 72]. Relevant to the present investigation, fasting whole blood was collected at baseline and end-of-study using standard phlebotomy procedures.

Blood samples were collected and processed according to previous reports [39, 73]. Briefly, peripheral blood mononuclear cells (PBMCs) were isolated by density centrifugation (Histo-paque; Sigma) and frozen at -80*C until further analysis. Upon thawing, cells were washed twice with phosphate buffered saline (PBS; Sigma) and stained with the following antibodies: TCR-Vd2 (FITC, Miltenyi Biotec) CD3 VioGreen, Miltenyi Biotec; Clone: REA613), ADRB2 (primary antibody Abnova; clone: 4A6C9; linked with lightning-link APC Labeling Kit,Expedeon), CD8 (VioBlue, Miltenyi Biotec; clone: BW135/80), CD4 (FITC, Miltenyi Biotec; clone: REA623; or PE-Cy5.5; ebioscience; clone:SK3), CD56 (APC-Vio 770; Miltenyi Biotec; Clone: REA196), CD20 PerCP-Vio 700; Miltenyi Biotec; clone: REA780), CD62L (PE, Miltenyi Biotec; clone: REA615), CD45RA (PE-Vio 770; clone: T6D11), CD45 (APC; Miltenyi Biotec; Clone: REA747), CD103 (APC, Miltenyi Biotec), CD31 (PerCP-Vio 770; Miltenyi Biotec; clone: REA730). Samples were stained at room temperature in the dark for 30 min. Samples were then washed and quantified via flow cytometry (MacsQuant, Miltenyi). Analysis of flow cytometric data was performed on FlowLogic (v7).

### Statistical procedures

Non normally distributed sets were log_10_ transformed and once again assess for normal distribution. Due to small sample size, within-subjects effects were calculated via a 2-tailed paired t-test in Microsoft Excel (v16.56) before fold changes were calculated to inform analyses from the ANOVA. Fold changes were calculated as (Post-Pre)/Pre. Hedge’s *g* was calculated to determine effect size of the small sample using the following equation in Microsoft Excel (V16.56): $\left(\frac{M1-M2}{SDpooled}\right)$. Absolute value of Hedge’s g is presented. Once fold changes were calculated, one-way ANOVA tests were conducted between HIIT, MICT, and UC groups for each cellular population. Pearson’s correlations were conducted to determine relationships between variables. The one-way ANOVA and Pearson’s correlation tests were conducted on GraphPad Prism (v8). *P*<0.05 was considered statistically significant. Statistical tests with *p*<0.1 are reported as trends. Data are presented as Mean+/-SD.

## Supplementary information

Additional file 1: **Supplementary Info Fig. 1.** No changes in circulating myokines with exercise training or between groups. No changes in the fold changes of (**A**) IL-7, (**B**) IL-15, (**C**) IL-6, or (**D**) Osteonectin were found between groups or within groups. Of note, there was a trend for a difference between the HIIT and UC groups in changes of IL-7 (*p*=0.14). Data are presented mean with *p<0.05.

## Data Availability

The data are available from the corresponding author on reasonable request.
