# Correction: Salutary effects of moderate but not high intensity aerobic exercise training on the frequency of peripheral T-cells associated with immunosenescence in older women at high risk of breast cancer: a randomized controlled trial

**DOI:** 10.1186/s12979-022-00276-x

**Published:** 2022-06-08

**Authors:** Grace M. Niemiro, Adriana M. Coletta, Nadia H. Agha, Preteesh Leo Mylabathula, Forrest L. Baker, Abenaa M. Brewster, Therese B. Bevers, Enrique Fuentes-Mattei, Karen Basen-Engquist, Emmanuel Katsanis, Susan C. Gilchrist, Richard J. Simpson

**Affiliations:** 1 Department of Pediatrics, The University of Arizona, Tucson, Arizona USA; 2 The University of Arizona Cancer Center, Tucson, Arizona USA; 3 Department of Health and Kinesiology, The University of Utah, Salt Lake City, Utah USA; 4 Cancer Control and Population Sciences Program, Huntsman Cancer Institute, Salt Lake City, Utah USA; 5 Department of Health and Human Performance, University of Houston, Houston, Texas USA; 6 School of Nutritional Sciences and Wellness, The University of Arizona, Tucson, Arizona USA; 7 Department of Clinical Cancer Prevention, The University of Texas MD Anderson Cancer Center, Houston, Texas USA; 8 Department of Radiation Oncology Clinical Research, The University of Texas MD Anderson Cancer Center, Houston, Texas USA; 9 Department of Behavioral Science, The University of Texas MD Anderson Cancer Center, Houston, Texas USA; 10 Department of Immunobiology, The University of Arizona, Tucson, Arizona USA; 11 Department of Cardiology, The University of Texas MD Anderson Cancer Center, Houston, Texas USA

**Correction to: Immun Ageing 19, 17 (2022).**

**https://doi.org/10.1186/s12979-022-00266-z**

Following publication of the original article [1], the authors reported an error on page 11. the URL “https://pubmed.ncbi.nlm.nih.gov/34302965/.” provided during proofing stage was captured as a normal URL. This should be corrected and captured as a reference “Simpson RJ, Boßlau TK, Weyh C, Niemiro GM, et al. Exercise and adrenergic regulation of immunity. Brain Behav Immun. 2021;97:303-318.”

The original article [1] has been updated.
