# Supplementary material for: Salutary effects of moderate but not high intensity aerobic exercise training on the frequency of peripheral T-cells associated with immunosenescence in older women at high risk of breast cancer: a randomized controlled trial
Source: Immun Ageing. 2022 Mar 23;19:17. doi: 10.1186/s12979-022-00266-z (PMC8941789; doi:10.1186/s12979-022-00266-z)
Supplement: Supplementary file 1 — Supplementary Info Fig. 1. No changes in circulating myokines with exercise training or between groups. No changes in the fold changes of (A) IL-7, (B) IL-15, (C) IL-6, or (D) Osteonectin were found between groups or within groups. Of note, there was a trend for a difference between the HIIT and UC groups in changes of IL-7 (p=0.14). Data are presented mean with *p<0.05. [file 12979_2022_266_MOESM1_ESM.docx]

We thank both reviewers for their thoughtful comments and constructive feedback on our manuscript. We have responded to each comment below and amended our manuscript accordingly. We hope that the reviewers will find our manuscript vastly improved from the original submission.

Reviewer #1: This study examined two different exercise training strategies and usual care in women with high risk for breast cancer. The study employed an expansive array of T cell phenotypes but used quite a modest sample size. The authors conclude that, in contrast to previous work, aerobic exercise alters circulating T cell distribution in a manner that HIIT shows evidence of greater senescent phenotypes while MICT displays a more favourable profile. The authors are to be commended for the comprehensive analysis using a longitudinal design. However, I have concerns regarding the analysis, both in terms of the statistics but also what appears to be a certain degree of redundancy in the results. Finally, the discussions and conclusions are reasonable, but the overall length seems excessive in places and fails to include discussions of previous work from the authors that appear to be highly relevant.

Specific comments

Abstract: "frequency" may not be the best term in conclusions, when cell number/concentration are predominantly reported.

We have changed this to ‘peripheral T-cell numbers’

P3 line 26, cross-sectional studies report a younger T cell profile. However, Simpson 2012 summarizes the longitudinal studies as well and reports minimal effects. This is an important omission, both in the intro and as part of the discussion. Importantly, why did this study succeed and others failed to improve T cells would be of value to the reader.

We have now mentioned in the introduction that there is a lack of consistency among longitudinal studies. Our assertion is that our study succeeded because: (i) our intervention was more rigorous in terms of being supervised and involving individualized training zone prescriptions; and (ii) our cohort were considered high risk of disease and were mostly obese as well as being older. Many previous studies have focused on healthy individuals that may have had less bandwidth for improvement after exercise training. We have alluded to this in the introduction and addressed it in more detail in the discussion.

Details regarding BCa risk would be better placed in methods vs. amidst the aims/purposes.

We agree and have removed these details from the introduction as they are already provided in the methods.

A central tenant to this ms is HIIT vs. MICT yet the rationale for this is missing from the introduction. There are arguments that exercise would improve immune function and thereby cancer risk, but why this population and why these modes of exercise? Stronger rationale is needed.

We agree and have added the following statement to our lead in:

“While there are reported health benefits of HIIT over MICT for several endpoints associated with cardiometabolic disease, few studies have determined the effects of HIIT on markers of immunity”.

We also included a recent reference of one known study that looked at immune related endpoints after HIIT.

These results are interesting and derived from a group with an excellent reputation in the field. My concerns are a large # of comparisons w/o any correction of the overall p values, an incomplete/misleading statistical analysis relative to what is being reported in tables and a small sample size that leaves the study underpowered to detect the key effects. Effect sizes are likely necessary to support these arguments and would fit within the current Tables.

We agree that the study is underpowered to complete certain analyses and we have acknowledged this in the discussion. Particularly, we are aware that we underpowered to detect group (3 levels) x time (2 levels) interaction effects, especially for endpoint measures that are known to have large levels of variability (numbers of peripheral T cell subsets) among individuals. Unfortunately, we are not in a position to recruit additional subjects for this study. We do agree that effect sizes will be important to bolster our interpretations/conclusions and have therefore added the Hedge’s *g* calculation to the tables. Hedge’s *g* is an indicator of effect size for small sample sizes (<20).

As described, the statistics were a between groups analysis on fold-change, which is shown in the Figures. The tables describe within group changes (time effects in text), which would only be examined with a significant interaction of group x time. This analysis is not included. As the ms is very similar to that from co-author Coletta's 2021 work in NK cells, why was the same analysis not used here?

We apologize that some of our described statistical procedures appear to be missing from the original manuscript. We have updated the manuscript to describe more clearly how the within and between subjects data points were analyzed. These are included in the materials and methods section under statistical procedures.

P13, line 23-25. Calling p=0.14 a trend is a stretch in this instance. Also, the word "trend" is used later (line 59) to describe the significant correlations. This is confusing use of the term.

We have removed this line from the manuscript.

With the Tables and the Figs, it appears that only the analyses were conducted on Figs. Are both of these necessary or could they be combined to reduce the number of outputs the reader is required to look through. With so many different comparisons, a p value correction may very well be in order as chance alone could be responsible for some of the effects. This needs to corrected for or an argument made why the reader should accept this approach.

Thank you for highlighting this oversight. We did complete paired analyses on the data presented in the table and we have now described these procedures in the methods section Our limited statistical approach is due to the small sample size, which we have acknowledged. While it would have been ideal to include more participants, we are no longer in a position to extend this study. Despite these limitations, we feel that the changes we observed here are worthy of publication, particularly because several prior studies have failed to document changes in numbers of peripheral T-cell subsets with exercise training.

The small sample size is acknowledged as a limitation, as is the lack of functional outcomes. Coupled with a quite modest sample size, these factors dampen enthusiasm overall. I would be interested to know why functional outcomes were not included, as it would have helped to counteract n=8/group.

We agree that several functional measure would have been ideal. Unfortunately, there was no specific funding support for the immune markers in this study so we were unable to complete comprehensive immune function endpoints.

The discussion seems overly long, particularly after a challenging results section. I would encourage the authors to carefully examine the discussion for ways to reduce the length to address key points more succinctly.

We have identified areas in the discussion that could be truncated and have tried to make these sections more succinct. We hope that this has improved the readability of our paper.

The figure image quality was quite low, making it difficult to read.

The journal apparently includes low resolution images in the PDF document that is downloadable for peer-review. However, there is a link on the PDF that allows access to the high-resolution version of the image.

Reviewer #2: General comments

The authors aimed to determined the effects of 12-weeks supervised exercise training on the frequency of T-cell subtypes in peripheral blood and their relationships with circulating levels of the muscle derived cytokines (i.e. 'myokines') IL-6, IL-7, IL-15 and osteonectin in older women at high risk of breast cancer. The intervention involved 3 sessions/week of either high intensity interval exercise (HIIT) or moderate intensity continuous exercise (MICT) and were compared to an untrained control (UC) group.

Specific comments

I have some concerns about the statistical analysis approach and results description.

1) The authors aimed to investigate the effect of two different interventions (HIIT and MICT) against a control group over 12-weeks on immunoscence in CD4 and CD8 T cells subtypes. In the assessment procedure, the authors described that fold changes were calculated for each cellular population. Does the one-way ANOVA was conducted to compare the fold-change of HIIT, MICT and control group, is it correct?

That is correct. Fold changes from pre to post intervention were determined and a one-way ANOVA was used to compare the fold changes across the three groups. This approach was taken due to the small sample size. We have provided a more detailed description of these procedures in our methods section.

2) In the tables, the authors described only time effect (PRE to POST intervention), does the one-way ANOVA was also conducted is it correct? Please, clarify this point.

We apologize for inadvertently omitting the within-subjects analysis procedures in the original version of our paper. We have now described these in our statistical analysis section and also added the Hedge’s *g* calculation to the tables.

3) In the results, the authors describe group effect of HIIT vs. MCIT "When looking at more mature lymphocyte subsets, only CD4 T cells were decreased in the HIIT group compared to the MICT group (Fig. 1E)", however there are no mention to p value. Please, clarify this point.

We have added the p values to these sentences.

4) So, authors presents the time effect (PRE to POST) and group effect (HIIT, MICT and control group) by multiples one-way ANOVA, while two-way repeated ANOVA present the time effect, groups effect and interactions in a accordance to the author's study design. Please, clarity this point.

We acknowledge that our study is underpowered to detect group (3 levels) x time (2 levels) interaction effects, especially for endpoint measures that are known to have large levels of variability (numbers of peripheral T cell subsets) among individuals. However, because we felt the data was worthy of being communicated through publication and we did not want to report potential type 2 errors, we elected to perform the within-subject analyses (i.e. time effects) and the between-subject effects separately. The within subject effects were detected using paired t-tests with Hedge’s *g* included as an indicator of effect size for small sample sizes (<20). The between subject differences were detected using one-way ANOVA to compare the delta values across the three groups.

5) Regarding the circulating myokines, it is very difficult to understand the figures due to the low quality of images.

The journal apparently includes low resolution images in the PDF document that is downloadable for peer-review. However, there is a link on the PDF that allows access to the high-resolution version of the image.

6) The authors presents the potential correlation of changes of myokines. There are difference between PRE to POST interventions in myokines?

There were no differences from pre to post intervention in the circulating levels of the measured cytokines at the study group level. This data was shown in our previous publication. (Coletta et al, 2021). In the present study, we were interested in whether or not changes in myokine levels at the individual level were associated with changes in the numbers of immune cells.
